# Challenges of Redeployment to ICU: A Qualitative Study Exploring Nurses’ Experience During the Coronavirus Pandemic

**DOI:** 10.1177/23779608251350221

**Published:** 2025-06-19

**Authors:** Barbara Whelan, Frank Fox, Jessica S. Hayes, Máire A. Connolly, Dympna Casey

**Affiliations:** 1 111805 School of Nursing and Midwifery, University of Galway, Galway, Ireland; 2 Global Health Programme, 37436 School of Health Sciences, University of Galway, Galway, Ireland

**Keywords:** Nurse redeployment, coronavirus pandemic, intensive care unit, qualitative study

## Abstract

**Introduction:**

The coronavirus pandemic placed significant stress on healthcare services globally, requiring rapid transformation to cope with critically ill patients. In many countries, including Ireland, staff redeployment to intensive care units was a core response.

**Objectives:**

To understand the experiences of nurses redeployed to intensive care units during the coronavirus pandemic and identify effective strategies for training and supporting nurses during future pandemics.

**Methods:**

A qualitative descriptive study using semistructured interviews with 19 nurses recruited from two hospitals in the west of Ireland. The sample consisted of senior nurses/clinical facilitators in intensive care units (n = 4), nurses previously in intensive care units and redeployed back (n = 2), and nurses redeployed from other clinical areas (n = 13). Data were collected between January 2023 and February 2024 and analyzed using thematic analysis.

**Results:**

Five main themes were identified: redeployment of staff to intensive care units; training; emotional impact; effective communication and valuing nurses; and planning for the next pandemic. Many nurses experienced significant stress and anxiety initially but felt more confident with support and training. Clear communication, effective training and strong support systems were important. Clinical facilitators provided just-in-time, hands-on training and reassurance, helping to make the experience of working in intensive care units less daunting. Training for those new to intensive care units focused pragmatically on what was “good-to-know” versus “essential-to-know.” Key findings highlight the importance of rapid, effective training, clear communication, and strong support systems, including psychological support.

**Conclusion:**

Redeployment to intensive care units during the coronavirus pandemic had a significant professional and emotional impact on nurses. Despite challenges, nurses demonstrated resilience and strong commitment to patient care. The findings emphasize the need for pandemic preparedness planning, including regular skills maintenance, effective communication strategies, and robust professional and psychological support during a health emergency. Recognizing and valuing nurses’ contributions is essential for fostering resilience, maintaining high quality clinical care and ensuring preparedness for future crises.

## Review of Literature

The coronavirus disease 2019 (COVID-19) pandemic placed extreme stress on healthcare systems globally as a result of a surge in positive cases and hospitalizations (Scott et al., 2023). Healthcare systems had to undergo rapid transformation in order to cope with the influx of critically ill patients, focusing on improving infrastructure, equipment, expertise, and increasing the availability of clinically trained staff (Veerapen & McKeown, 2021). The pandemic highlighted the necessity and demand for critical care capacity and proper planning for these services (Mer et al., 2022). Various approaches were implemented to ease the burden on intensive care units (ICUs), including transforming noncritical care beds into temporary ICU spaces (Grasselli et al., 2020) and transferring critically ill patients between hospitals (Painvin et al., 2021). A key limiting resource globally was the availability of clinically trained staff which was exacerbated by healthcare workers contracting the virus and subsequently being unavailable for work. Redeployment of staff to ICUs and critical care units became a core element of the response in many countries including Ireland (Ryder et al., 2022).

Healthcare workers faced numerous anxieties at the start of the pandemic, including concerns about obtaining adequate personal protective equipment (PPE), contracting COVID-19 at work and infecting family members at home, concerns about their family's needs, the challenge of delivering competent care in unfamiliar clinical areas and a shortage of information and effective timely communication (Shanafelt et al., 2020). These fears were compounded for redeployed nursing staff who had to rapidly adapt to a high-stress environment and learn new skills (Kennedy et al., 2022). Induction and training of redeployed nursing staff was key to successful redeployment (Vera San Juan et al., 2022). Rapid upskill training programs were developed to equip nursing staff for deployment to ICU (Causby et al., 2024). The training varied in length and content, focusing on essential skills such as mechanical ventilation, infection prevention and control, patient monitoring and proning (Brickman et al., 2020; Camilleri et al., 2022; Engberg et al., 2021; Yuriditsky et al., 2021). Effective training combined theoretical sessions with practical, simulation-based (Brickman et al., 2020; Camilleri et al., 2022; Engberg et al., 2021; Naik et al., 2020; Sela et al., 2020; Yuriditsky et al., 2021) or virtual reality training (Lee & Han, 2022), catering to the diverse learning needs and previous experiences of the nurses.

At the start of the COVID-19 pandemic, with only 256 ICU/High Dependency Unit (HDU) beds, equating to 5.2 ICU/HDU beds per 100,000 population, Ireland's ICU capacity was already significantly below the Organisation for Economic Co-operation and Development (OECD) average of 12 ICU beds/100,000 people (National Clinical Programme for Critical Care, 2021). Furthermore the main limiting factor in increasing critical care capacity was the lack of experienced ICU nurses (National Office of Clinical Audit, 2021). At the outset of the pandemic the Health Service Executive, responsible for the management and delivery of health and social care services in Ireland, responded by redeploying nurses to critical care, guided by national recommendations (Health Service Executive, 2020). These recommendations emphasized redeploying nurses with previous ICU experience and those with experience and competencies comparable to critical care (e.g., coronary care, surgical theater, anesthesia, recovery, postanesthetics care) (Health Service Executive, 2020). Recognizing the importance of training, the recommendations called for tailored educational programs to address gaps in competency for redeployed nurses.

Understanding the experience and impact of the COVID-19 pandemic on redeployed nurses is important so that we can learn how best to support them to deliver effective care during health crises (Causby et al., 2024). It is also important that we understand the impact of redeployment on nurses to gain greater knowledge for workforce planning for future pandemics (Ryder et al., 2022). This is even more pertinent given that experts now agree that the probability of a future influenza pandemic is high (Dzau & Yadav, 2023). This study explored nurses’ experience of redeployment to ICU during the pandemic and the training that they received, culminating in recommendations for redeploying nursing staff in future pandemics. To our knowledge, this article is the first to capture the experience of nurses within Ireland who were redeployed to work in ICU during the pandemic.

## Methods

### Design

A qualitative descriptive study was conducted using semistructured individual interviews with nurses redeployed to ICU during the COVID-19 pandemic. Some nurses had previous ICU experience, while others had none. The study also included senior personnel and clinical facilitators involved in redeployment planning.

### Setting

Participants were recruited from two public hospitals in the west of Ireland, both providing critical care, including ICU and HDU beds. Both hospitals served as referral centers for COVID-19 cases during the pandemic.

### Population and Sampling

We used purposive and snowball sampling to identify interview participants. Initially, purposive sampling identified information-rich cases with varied experiences. As the study progressed, we also used snowball sampling whereby participants referred colleagues who met the study's inclusion criteria and could provide additional insights.

Two groups of participants were recruited for this study: redeployed nursing staff and senior personnel or clinical facilitators. Redeployed nursing staff were eligible to participate if they had been redeployed to work in ICU during the COVID-19 pandemic, had not previously worked in ICU or had not done so for over 1 year, and had received training in mechanical ventilation during the pandemic. Participants were required to be aged 18 years or over and willing to provide informed consent. Nurses who had worked in ICU in the year prior to the interview were excluded. Senior personnel or clinical facilitators were eligible if they had been directly involved in the process of redeploying nursing staff to ICU during the pandemic, were aged 18 years or over, and provided informed consent. Staff not involved in redeployment decisions or processes were excluded.

All participants received an information sheet about the study and had the opportunity to ask questions before participating. A total of 19 nurses were interviewed.

### Data Collection

Data were collected between January 2023 and February 2024 by one researcher (BW). Potential interviewees were approached by a clinical facilitator/assistant director of nursing or informed through meetings. Some nurses referred colleagues who consented to be contacted by the researcher. All participants received an information sheet and consent form and were given the opportunity to ask questions about the study before taking part. All participants signed a consent form.

Semistructured, individual interviews explored ICU redeployment experience, training and preparation for redeployed nurses, experiences in ICU, and pandemic preparedness. The researcher (BW) was a postdoctoral researcher on the team with training and experience in conducting interviews, who did not have any relationship with the participants prior to the interviews. All personal identifiers from the transcripts were removed and interviewees were grouped in generic role categories to avoid individuals being identifiable in interview quotes which are presented in the results below.

Interviews were arranged at a time and place that was convenient to the participants and were held in person (n = 7), online through zoom (n = 5), or by telephone (n = 7). All interviews were audiorecorded with the permission of the participants. The average time of the interviews was 36 min with a range of 12 to 78 min. Data collection continued until data saturation was reached and no new themes were identified.

### Data Analysis

Interviews were transcribed *verbatim* and transcripts were checked to ensure the accuracy of the transcription. Data analytics was undertaken by two members of the research team (BW and DC) and followed the guidelines of thematic analysis by Braun and Clarke (2006). Initially, all of the transcripts were read by BW to ensure familiarization with the content. Initial codes were generated by systematically highlighting the raw interview data and these codes were then collated into potential themes, which were reviewed and refined in an iterative process. This stage was reviewed by DC who checked the themes against the data to ensure they accurately represented the information. A mind-map of the themes was produced to help visualize relationships and patterns among the themes. Themes were further examined and refined during discussions between BW, DC, and the other authors. The final themes were then prepared for reporting, including participant's quotes, with anonymity maintained. The software package, NVivo 13/R1/2020 (QSR International Melbourne, Australia) was used to enable data organization and retrieval. Thick description, whereby a detailed description of the research context and the characteristics of the participants, enhanced both credibility and transferability. Participants were purposively sampled for maximum variation to ensure a variety of experiences and responses. Maintaining proximity to the original data, by presenting direct quotes, ensured an accurate portrayal of the participants’ perspectives, supporting conformability, and credibility (Lincoln & Guba, 1985). The study adhered to the Standards for Reporting Qualitative Research (O'Brien et al., 2014).

### Ethical Considerations

Ethical approval for the study was given by Galway Clinical Research Ethics Committee (Ref: C.A 2868), and permission was obtained for both participating hospitals. All participants were aged 18 years or older and were fully informed about the purpose and nature of the study prior to participation, both with an information letter and verbally. Written informed consent was obtained from each participant, and they were advised that their involvement was entirely voluntary, with the right to withdraw at any point without penalty or consequence.

To protect anonymity and confidentiality, all data were de-identified during transcription, with participants assigned codes and a unique number. No identifying details were included in the final transcripts. Audio recordings were stored securely on a password-protected, encrypted device accessible only to the research team. Once transcribed, audio files were securely deleted. Transcripts were stored on a secure server and managed in accordance with institutional data protection policies.

## Results

### Sample Characteristics

In total 19 nurses were interviewed, of which all, with the exception of one, were female. Four were senior nurses/clinical facilitators in ICU, two were nurses who had been ICU nurses for 12 and 34 years, respectively, and were redeployed back to ICU, 13 were nurses who were working in other clinical areas such as theater and endoscopy and who were redeployed to ICU. The average years of experience working as a nurse was 25 (range 6–40 years).

**Figure 1.**
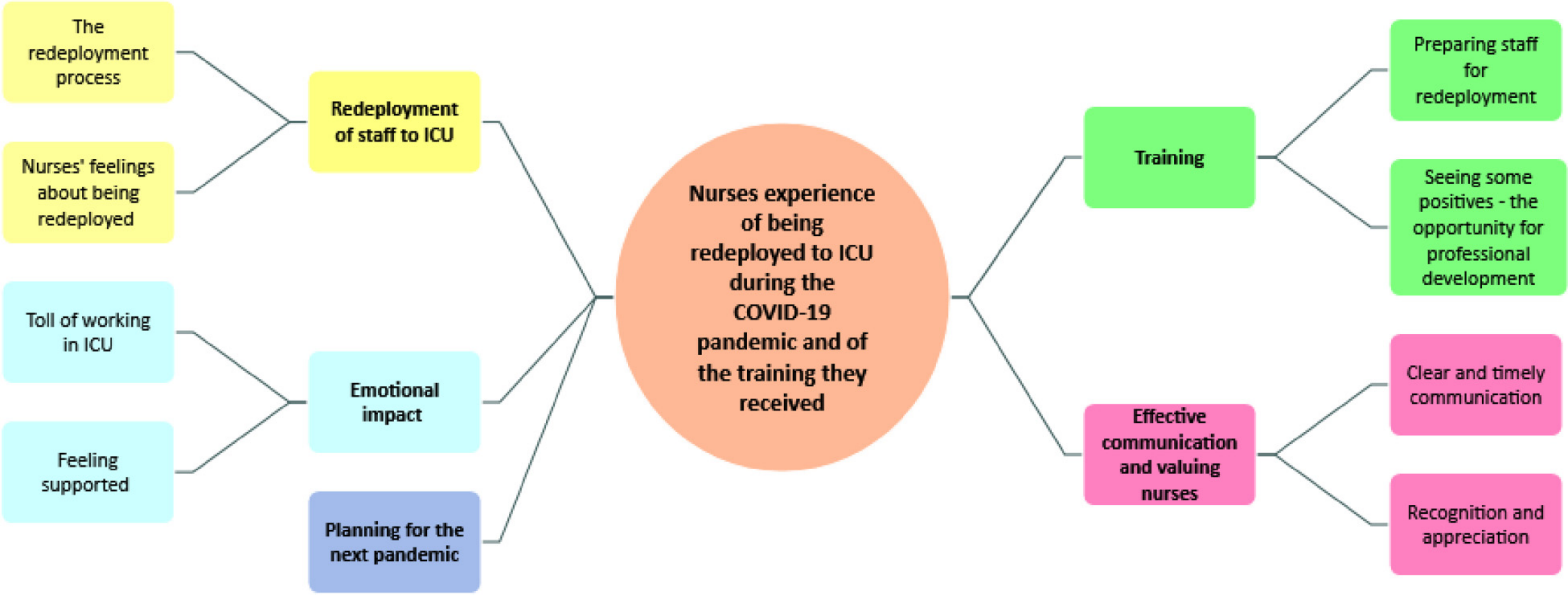
A mind map of the major themes and subthemes.

Five main themes with subthemes were identified. Figure 1 presents a mind map of the major themes and subthemes and a description for each is presented below. Direct quotations from the interviews are also presented with the speaker identified with a code (PHCP) and unique number (1–19). No further information is provided in order to protect anonymity.

## Theme 1: Redeployment of Staff to ICU

This theme explores the process and implications of redeploying staff to ICU, including who was redeployed, how they were informed, their roles in ICU and their feelings about being redeployment.

### The Redeployment Process

Hospitals followed the national surge plan to increase ICU nursing capacity. This involved redeploying staff from clinical areas with relevant competencies, such as theater and anesthesia and bringing back nurses who had previously worked in ICU. Those who had previously been ICU nurses, were treated as ICU nursing staff. Those without ICU experience served in support roles such as runners on corridors, checking their colleagues’ PPE, assisting in ICU, the postanesthesia care unit (PACU) and the high dependency unit (HDU) and helping with proning patients.

“*Again it would be running around getting ventilators, dialysis equipment, pillows, pillows, pillows, pillows … I was constantly fetching things and equipment.”* (PHCP10)

The two hospitals took different approaches to redeployment. One required all theater nurses, except for a minority that got dispensation due to their age/health/family issues, to work some shifts in ICU. The other selected a discrete group, including theater nurses and those from temporarily closed units like endoscopy and cardiac rehab. Redeployed nurses were allocated to different areas by ICU managers and worked there for a few weeks during surges before returning to their usual roles. They typically worked for two or three stints, each lasting approximately 4 to 6 weeks throughout the pandemic. Every effort was made to assign less critically ill patients to the redeployed nurses. On the rare occasions, when a redeployed nurse had to care for a ventilated patient, oversight and support from an ICU nurse was provided.

The manner in which nurses were informed and communicated about their redeployment varied, with some nurses receiving e-mails or phone calls, while others were informed face-to-face in person or during staff meetings. Nurses who were not informed face-to-face and in person expressed a preference for this more personal approach.

“*An e-mail was sent and we were directed to return to ICU by the Assistant Director of Nursing … I suppose it would have been nicer if you were asked in person rather than e-mails.”* (PHCP4)

### Nurses’ Feelings About Being Redeployed

Many nurses felt scared, terrified, and overwhelmed by redeployment.

“*It was daunting and it was a bit nerve-racking because I had never worked in ICU.”* (PHCP13)

For some, personal circumstances such as having vulnerable family members in at-risk groups for COVID-19, heightened their stress. This stress was magnified by the lack of information about the disease and the media portraying a “poor outcome if you caught it” (PHCP7).

Some nurses felt useful and recognized their contribution to supporting their colleagues in ICU, while others felt superfluous, describing themselves as a “*spare tool*” (PHCP8).

*“At the beginning … I think most of us felt like lemons. We didn’t know where things were.”* (PHCP16)

Over time, many nurses found ICU work less stressful as they gained experience and felt better prepared for what was expected of them. However, some theater nurses initially felt isolated and stigmatized by colleagues who were not redeployed, creating conflict, and hurt.

“*We were literally treated like lepers when we were there. We weren’t allowed to get changed with the theatre staff… We weren’t allowed to use their scrubs.”* (PHCP14)

Equally, nurses expressed frustration over perceived inequalities in redeployment, questioning why experienced ICU nurses were assigned to less critical roles like vaccinations.

“*… I recognised three people who had been very senior ICU nurses who had gone into management and who were now giving vaccinations. And I thought why are they giving vaccinations and I’m up in ICU trying not to kill anyone. This seems unfair.”* (PHCP9)

Nurses with previous ICU experience did not find redeployment as overwhelming.

“*I was never afraid. I never didn’t want to go into work or disliked being in there. No, I loved every second of it.”* (PHCP12)

## Theme 2: Training

This theme describes the training provided to redeployed staff, covering how training was delivered and the topics addressed for nurses with and without previous experience of ICU. It also discusses the role of the clinical facilitators in training and the professional development opportunities provided by redeployment.

### Preparing Staff for Redeployment

For those with prior ICU experience, training focused on changes in practice in ICU due to COVID-19 such as how to prone a patient and put an oxygen-hood on for noninvasive ventilation. For nurses without ICU experience, clinical facilitators prioritized essential knowledge and patient safety.

“*We're not teaching these nurses to be critical care nurses, critically thinking at the bedside, we're teaching them to be safe at the bedside.”* (PHCP1)

Nurses were given clinical parameters to monitor such as blood oxygen levels, with clear instructions to seek help if thresholds were crossed. Efforts were made to avoid overwhelming nurses with information, ensuring they felt comfortable with what they were being asked to do, so they would not be fearful of coming back to ICU to do another shift. Some nurses received information packs with routine ICU checks and competency skills, which were useful for identifying training needs. In addition, laminated pages in ICU at the bed space provided quick references for critical procedures. Nurses liked “hands-on” training which they found more effective than videos or prescheduled sessions. The clinical facilitators played a key role in providing “ad hoc” training, especially for nurses needing a recap or training on new equipment.

“*And that was the biggest help was the facilitator. Now if there wasn’t a facilitator there I think it would be more daunting to be quite honest with you.”* (PHCP12)

While many nurses were happy with their training, some felt that they needed more.

“*… the training that we got was appalling really… Going up to ICU then to be honest with you I only had about an hour's training… Yeah an hour or two at the max.”* (PHCP11)

As previously noted, the two hospitals in this study, adopted different approaches to staff redeployment which impacted the effectiveness of training. In one hospital, a large pool of theater nurses were available for redeployment, resulting in an inconsistency in who was being redeployed to ICU on a daily/weekly basis. In contrast, the other hospital redeployed a smaller, select group of nurses, allowing for them to consistently apply what they learned in ICU. This issue was highlighted by both a clinical facilitator and some of the redeployed nursing staff.

“*… the problem was they were sending up different nurses every day… So I used to take them off, but I was teaching basic assessment regularly every single day to different people, every single day. So by year two that was something I really learned, don’t send me different people every day, if you send me ten people for one month that would be better than sixty people different every week.”* (PHCP2)

*“…You know what if they actually trained a few of us up properly then we would be useful maybe to take a HDU type patient.”* (PHCP8)

Seeing some positives—the opportunity for professional development.

Being deployed to work in ICU provided nurses with the opportunity for professional development. Some discussed how they gained new clinical skills and a better understanding of critical care.

“*I actually learned a lot in there to be quite honest. I learned how to do arterial blood gases and blood gases and all that sort of thing, basic stuff that I would never have done anyway.”* (PHCP14)

The opportunity to work in ICU was also perceived as an opportunity to step out of one's comfort zone, to experience a different clinical area and to work with new colleagues.

“*I think it's quite good for everyone at some point in their career as such to be taken out of their comfort zone. I think it shakes you up a little bit and stops you being a bit complacent. And to see what can go wrong and what people can be like.”* (PHCP16)

## Theme 3: Emotional Impact

This theme explores the emotional impact of redeployment on nurses, how they managed stress and the support they received from ICU colleagues and their own peer group.

### Toll of Working in ICU

The transition to working in ICU, where patients were critically ill and there were many unknowns about the SARS-CoV-2 virus, working in a new clinical environment and with new colleagues, all took an emotional toll on nurses. Fear was amplified by uncertainty about how the virus was transmitted and increased exposure to COVID-19 patients.

“*You’d be petrified we’ll say after ICU. I never showered so much in all my life… I showered myself so much because I was petrified in case I would give it to my husband you know what I mean or anyone else that was around me you know because you just didn’t know.”* (PHCP11)

Some nurses felt additional pressure from family members who didn't understand why they would risk working in ICU, leading to stress and a sense of isolation. The stress of redeployment led some nurses to feel they couldn't endure it again, with one nurse (PHCP11) expressing how she would “*leave the nursing profession”* if faced with it again. For others the stress was heightened by having to work in a new clinical environment where they did not feel prepared or particularly useful. Sometimes these concerns were disregarded by management.

“*It was the not knowing and it was especially for experienced people to feel stupid all the time through no fault in my experience of the staff up there… The fact that it wasn’t necessary … it was raised that maybe we’re not needed up there [in ICU] the response that came back [from management] was well if you’re not needed there we’ll just send you to one of the wards … That's kind of contempt for what we’re trying to say …That all adds into the stress of it.”* (PHCP9)

### Feeling Supported

Despite the challenges, support from permanent ICU staff and clinical facilitators was crucial. Redeployed nurses were relieved when expectations regarding their role were set and recognized as being a support role only.

“*They didn’t put too much pressure on us. They knew from the get-go that we were there to assist really.”* (PHCP10)

The clinical facilitators played an essential role in supporting redeployed nurses, providing reassurance and guidance.

*“The clinical facilitator was very good. She’d come in every day and she asked us and she’d go over things with us you know. Every day I was on she’d go over different things and we could ask things.”* (PHCP18)

In one hospital, the redeployed nurses used a WhatsApp group for support which allowed them to check who would be on the same shift and to share information about the ICU situation. Many nurses felt they needed counseling after their redeployment but were disappointed by the lack of support available. Management seemed to expect them to move on and get back to their old jobs, which were very busy due to a backlog of elective surgeries.

*“I felt as if there should have been a bit more support for us… You know and maybe a bit more counselling sessions for us or something I think.”* (PHCP11)

## Theme 4: Effective Communication and Valuing Nurses

This theme highlights the importance of clear communication and recognition for nurses during their redeployment to ICU. It explores the need for nurses to receive timely information about their roles, new policies, and the COVID-19 situation in the hospital.

### Clear and Timely Communication

Clear and timely communication from hospital management was important for keeping nurses informed about their roles, new policies, and updates about the pandemic. Nurses wanted to know the number of COVID-19 patients in ICU, which gave them a sense of how things were progressing and what they might face. Because of the changing nature of the pandemic and better understanding of the SARS-CoV-2 virus, policies were changing regularly with the hospital but were sometimes not communicated well to the nurses which caused frustration and a loss of morale.

“*The changes were going to be inevitable as you learn more but the communication just wasn’t there really. And on any day if you asked ten people the same question you could get five or six different answers. So I mean again from the management that communication wasn’t great. It was very poor.”* (PHCP9)

### Recognition and Appreciation

Senior staff and clinical facilitators acknowledged the crucial support role that redeployed nurses played in ICU.

*“I don't think we could have managed the first busy weeks of COVID when it hit, peaked those peak periods without the theatre staff because they were like runners, or they were like a nurse who would stay at the end of the bed. And just make sure everything was okay.”* (PHCP19)

Some redeployed nurses felt recognized and appreciated by ICU staff and management, which motivated them.

*“I found we were so appreciated and I have to say the ward sister was very good there [in ICU] and very encouraging. And afterwards she came back with presents to us and cards.”* (PHCP13)

This contrasted with the lack of acknowledgement many felt from senior management and their own department managers.

“*The manager of ICU was very good. We didn’t see much from theatre at all management wise. A little pat on the back once in a while. The manager in ICU really looked after us and then when we went back to theatre I think we got a mention in a newsletter or something and another pat on the back.”* (PHCP16)

## Planning for the Next Pandemic

Participants gave suggestions for how surge capacity in ICU could be increased if there were another pandemic. Suggestions included selected nurses doing rotations to ICU in “peacetime” or having regular teaching sessions on critical care clinical skills. However, on a practical note, some nurses felt that it would not be possible to do this because clinical areas are already so understaffed, and they could not afford the time. Unsurprisingly, some participants could not bear to think of there being another pandemic with two nurses suggesting that they would leave the nursing profession or refuse rather than go through the same experience of redeployment again.

“*I think if a pandemic started next week and we were told we’re going to ICU there would be mass sick leave, … a lot of people who were here would just not put themselves through it again.”* (PHCP9)

In addition, some nurses felt that because the sacrifices that the redeployed nurses made were not adequately acknowledged, they questioned why these workers would take such risks again if there were another crisis.

“*I don’t think that they* [the redeployed nurses] *got valued enough either from what they had to contribute at the time, by the health service, by anybody. There was no good recognition of their value.”* (PHCP19)

Finally, many nurses discussed how redeployment needed to be fair and those with skills which could best fit ICU should be called upon first. Also, some nurses highlighted the need for flexibility in shift times for redeployed nurses, proper orientation to ICU, easy access to the clinical facilitators and support services available for nurses who have been negatively impacted by their experience in ICU.

## Discussion

This study highlights the importance of rapid training, clear and effective communication and strong support systems in order to address the challenges faced by redeployed nursing staff. Despite high levels of stress and anxiety, many of the nurses in our study showed resilience and high levels of commitment to patient care throughout the pandemic. The study illustrates the emotional and professional impact of redeployment on nursing staff during the COVID-19 pandemic. The national surge plan to increase ICU capacity (Health Service Executive, 2020) depended on the flexibility and resilience of nursing staff who were willing to be moved from their clinical areas of expertise to meet the urgent demands of ICU. The redeployed nurses took on various roles within ICU ranging from direct patient care for those with prior ICU experience to support roles for those less familiar with the ICU environment.

The plan for redeployment was often not communicated well to nursing staff with it being perceived as being impersonal and unclear as to why particular nursing staff were chosen for redeployment over others. Nurses would have liked clear, more respectful, and direct communication from managers about the plan for redeployment as this may have lessened their concerns and helped them to understand the rationale for why they were chosen for redeployment. Previous research supports the need for staff involvement in pandemic preparedness strategies to reduce anxiety and improve morale (Ballantyne & Achour, 2022; Doyle et al., 2022). Communication is critical during a pandemic response (Fernandez et al., 2020; Shanafelt et al., 2020; Vera San Juan et al., 2022) and our study highlighted its importance for nurses during their redeployment to ICU, along with the need to feel valued (Shanafelt et al., 2020). Providing clear communication and accurate data about COVID-19 admissions was vital to reduce uncertainty and rumors related to redeployment (Panda et al., 2021). Recognition and appreciation from ICU staff and management motivated the redeployed nurses by acknowledging their efforts and sacrifices. Gestures such as personal thanks, gifts and cards were appreciated. This was in contrast to the experience some nurses had in their original departments, where there was a noticeable lack of recognition from management, causing them to feel undervalued. As with findings from other research (Ballantyne & Achour, 2022), our study showed that nurses wanted senior managers to acknowledge the challenges they faced in being redeployed.

Nurses’ initial fears and anxieties about being redeployed were driven not only by the newness of working in the ICU environment but also by concerns for personal safety and the wellbeing of their families. The fear of getting COVID-19 was not unfounded with healthcare workers at greater risk of contracting COVID-19 as 10% of all diagnosed cases in Ireland in the early pandemic months were nurses (Irish Nurses and Midwives Organisation, 2020). This fear and anxiety was compounded by some nurses feeling pressure from family members to stop working in ICU and contributed to feelings of isolation at home and in the community. This was further exacerbated by a division between redeployed nurses and their colleagues in other departments, who were hesitant to interact with them due to fear of infection and this amplified the feeling of isolation experienced by the redeployed staff. These feelings evolved overtime for many of the nurses as supportive and welcoming ICU colleagues, along with the opportunity to ask questions, significantly eased their transition, especially for those redeployed as part of a small group with regular ICU shifts. Bergman et al. (2021) found that both ICU nurses and those redeployed valued mutual support, with ICU nurses appreciating the help from redeployed colleagues. Doyle et al. (2022) described the importance of the “camaraderie of pandemic working” in counteracting the negative effect of redeployment on morale, emphasizing that redeployed staff need to feel supported within their capabilities to foster a positive experience. Our study points to the need for support strategies that recognize the role of the redeployed nurses, making sure they feel valued and part of a team.

Training for redeployed ICU nurses was essential for equipping them with the necessary skills. The main education priorities for hospitals in general, in Ireland, were infection prevention and control, PPE use, critical care skills and briefings on updated documentation (Ryder et al., 2022). In our study, the approach to training for those who had never previously worked in the ICU environment was pragmatic and focused on what was “good-to-know” versus “essential-to-know.” The clinical facilitators, whose role it is to support nurses in developing clinical skills and competencies, played a critical role in providing just-in-time training and support and this has been recognized in previous research (Kennedy et al., 2022; Ryder et al., 2022). Hands-on and “on-the-job” training were favored by nurses, reflecting a preference for learning through direct experience rather than theoretical instruction (Causby et al., 2024; Ryder et al., 2022). Similar to other research studies, there was very little enthusiasm among nurses for online training (Causby et al., 2024). The focus on practical, experiential learning was important in building nurses’ confidence and in enabling them to adapt more quickly to their roles in ICU. Despite many nurses being happy, overall, with the training that they received, some nurses felt underprepared, highlighting the challenge of adequately training large numbers during a crisis.

Despite redeployment challenges, nurses identified positive outcomes such as professional development and exposure to new clinical areas, aligning with previous research (Danielis et al., 2021; Doyle et al., 2022). Many saw redeployment as an opportunity to build clinical skills, resilience, and versatility. Informal peer support, including WhatsApp groups, helped nurses share information, offer encouragement, and foster a sense of shared purpose (Vincent et al., 2022). However, transitioning back to original roles was difficult due to a lack of formal counseling and the psychological toll of the pandemic. Some nurses questioned their ability to face similar redeployment again in the future, with a few considering leaving the profession or taking sick leave, if it arose. This highlights the need for reflective opportunities to build positive narratives from these experiences (Greenberg et al., 2020). The gap in support was compounded by postpandemic workloads from delayed regular or nonpandemic care. Providing psychological support for healthcare workers, including varied options such as counseling and morale-enhancing activities, is strongly recommended (Aziz et al., 2020; Conti et al., 2020; Greenberg et al., 2020; Xu et al., 2021; Mer et al., 2022).

Participants emphasized the need for readiness and ongoing education to boost ICU surge capacity for future pandemics. Suggestions included “peacetime” rotations and targeted teaching sessions to enhance skills and knowledge sharing, reducing the need for crisis training (Kennedy et al., 2022). This approach fosters emergency preparedness and continuous professional development. However, challenges such as nursing shortages and the psychological toll of COVID-19 were noted, with some nurses questioning their ability to face similar redeployment. Managers must ensure time for reflection and learning to transform these experiences into growth opportunities (Greenberg et al., 2020). Transparent, fair redeployment strategies were also advocated, prioritizing those with ICU-relevant skills.

## Strengths and Limitations

To the authors’ knowledge this is the first study that focused on redeployed nurses’ experience of working in ICU in Ireland during the pandemic, offering valuable insights for future crises. Perspectives from a range of healthcare workers provided a comprehensive view and because the interviews took place more than 3 years after the start of the pandemic it meant that the nurses had time to assimilate their experience of redeployment. However, because it was conducted retrospectively when the heightened fear of the initial pandemic was over, it may also have impacted recall.

The findings of this study have several important implications for nursing practice, which are discussed in detail in the following section.

## Implications for Practice

The findings from this study were used to inform seven key recommendations for consideration when redeploying nursing staff to ICU during any future pandemic.

- *Ensure clear policy, decision making and communication between management and staff on redeployment process*—Prioritize timely, consistent, and transparent communication regarding redeployment policies, training schedules, and role expectations to minimize uncertainty and stress. Provide regular briefings on the number of COVID-19 patients in ICU to help nurses understand the situation and prepare for their shifts. Ensure that management is transparent about who is being redeployed and why they are being chosen. In addition, implement a feedback system where redeployed nurses can share their experiences, which management can use to address concerns and improve processes in real time.
- *Provide targeted skill-based training*—Focus training programs on essential critical care skills, tailored to nurses’ existing knowledge levels to ensure it is relevant and not overwhelming. Online training could be used for theoretical knowledge but should be kept to a minimum for nurses who have never worked in ICU and it should always be complemented with hands-on sessions where nurses have an opportunity to apply what they have learned in controlled, supervised settings.
- *Provide emotional and psychological support to redeployed nurses*—Recognize the emotional toll on redeployed nurses and monitor their mental health. Provide comprehensive support systems including counseling and debriefing sessions, which should also be available once they return to their original posts. Ensure permanent ICU staff are positively prepared to receive redeployed staff and are welcoming and supportive towards them.
- *Prioritize longer duration of redeployment*—Redeploy smaller numbers of the same nurses for longer periods of time rather than day-to-day ad hoc redeployment from a large pool of nurses.
- *Give recognition and appreciation*—Regularly acknowledge the efforts and sacrifices of redeployed staff through formal recognitions and informal appreciations to boost morale and validate their critical contributions.
- *Recognize and build on the role of clinical facilitators in training and supporting redeployed nurses*—Ensure a sufficient number of clinical facilitators to provide hands-on training and support to redeployed nurses.
- *Establish pandemic preparedness training programs*—Establish regular training programs for a specific number of nurses who can subsequently be redeployed to ICU so that they are familiar with ICU procedures and environments. This could be part of a national pandemic preparedness training program to include short rotations in ICU during normal nonpandemic periods to ensure that they have an optimal chance of retaining the learnt critical care skills and thereby reduce the learning curve during health emergencies. Schedule regular refresher courses that keep all nurses updated on the latest ICU care standards and technologies, ensuring that their skills remain relevant and can be rapidly deployed.

## Conclusion

This study adds to the body of knowledge concerning nurse redeployment during a pandemic, emphasizing the need to learn from these experiences to better prepare for future pandemics. Redeployment to ICU during the COVID-19 pandemic had a significant emotional and professional impact on nurses. Key findings highlight the importance of rapid, effective training, clear communication, and strong support systems, including psychological support. Clinical facilitators played an important role in providing just-in-time, hands-on training and reassurance, helping to make the experience of working in ICU less daunting. Training was pragmatic, focusing on what was “good-to-know” versus “essential-to-know” with a preference among nurses for practical learning. Despite the challenges, nurses demonstrated resilience and commitment to patient care. The findings emphasize the need for a proactive approach to pandemic preparedness, including regular skills maintenance, effective communication and acknowledgment of nurses’ contributions during crises such as pandemics. Further research is needed to define the numbers of nurses required for a pandemic preparedness ICU training program, the duration, frequency of training, and learning outcomes.
